# Directed evolution of a β-mannanase from *Rhizomucor miehei* to improve catalytic activity in acidic and thermophilic conditions

**DOI:** 10.1186/s13068-017-0833-x

**Published:** 2017-06-02

**Authors:** Yan-xiao Li, Ping Yi, Qiao-juan Yan, Zhen Qin, Xue-qiang Liu, Zheng-qiang Jiang

**Affiliations:** 1 Beijing Advanced Innovation Center for Food Nutrition and Human Health, Bioresource Utilization Laboratory, College of Engineering, China Agricultural University, No. 17 Qinghua Donglu, Haidian District, Post Box 294, Beijing, 100083 China; 2 Beijing Advanced Innovation Center for Food Nutrition and Human Health, College of Food Science and Nutritional Engineering, China Agricultural University, Beijing, China

**Keywords:** Directed evolution, *Rhizomucor miehei*, β-Mannanase, Acidic pH, Optimal temperature, Biorefinery

## Abstract

**Background:**

β-Mannanase randomly cleaves the β-1,4-linked mannan backbone of hemicellulose, which plays the most important role in the enzymatic degradation of mannan. Although the industrial applications of β-mannanase have tremendously expanded in recent years, the wild-type β-mannanases are still defective for some industries. The glycoside hydrolase (GH) family 5 β-mannanase (*Rm*Man5A) from *Rhizomucor miehei* shows many outstanding properties, such as high specific activity and hydrolysis property. However, owing to the low catalytic activity in acidic and thermophilic conditions, the application of *Rm*Man5A to the biorefinery of mannan biomasses is severely limited.

**Results:**

To overcome the limitation, *Rm*Man5A was successfully engineered by directed evolution. Through two rounds of screening, a mutated β-mannanase (m*Rm*Man5A) with high catalytic activity in acidic and thermophilic conditions was obtained, and then characterized. The mutant displayed maximal activity at pH 4.5 and 65 °C, corresponding to acidic shift of 2.5 units in optimal pH and increase by 10 °C in optimal temperature. The catalytic efficiencies (*k*_cat_/*K*_m_) of m*Rm*Man5A towards many mannan substrates were enhanced more than threefold in acidic and thermophilic conditions. Meanwhile, the high specific activity and excellent hydrolysis property of *Rm*Man5A were inherited by the mutant m*Rm*Man5A after directed evolution. According to the result of sequence analysis, three amino acid residues were substituted in m*Rm*Man5A, namely Tyr233His, Lys264Met, and Asn343Ser. To identify the function of each substitution, four site-directed mutations (Tyr233His, Lys264Met, Asn343Ser, and Tyr233His/Lys264Met) were subsequently generated, and the substitutions at Tyr233 and Lys264 were found to be the main reason for the changes of m*Rm*Man5A.

**Conclusions:**

Through directed evolution of *Rm*Man5A, two key amino acid residues that controlled its catalytic efficiency under acidic and thermophilic conditions were identified. Information about the structure–function relationship of GH family 5 β-mannanase was acquired, which could be used for modifying β-mannanases to enhance the feasibility in industrial application, especially in biorefinery process. This is the first report on a β-mannanase from zygomycete engineered by directed evolution.

**Electronic supplementary material:**

The online version of this article (doi:10.1186/s13068-017-0833-x) contains supplementary material, which is available to authorized users.

## Background

As the huge consumption of fossil fuels results in enormous emission of greenhouse gases, more and more attention is focused on the consequent air pollution and global warming [1]. To overcome this problem, biorefinery tends to be a potential method to produce biofuel from renewable cellulose and hemicellulose, which is a green alternative to fossil fuels [2]. As one of the major constituents of hemicellulose, mannans exist in many biomasses, such as coffee ground, copra meal, palm kernel cake, and some plant gums [3]. In biorefinery process, polysaccharides in biomass should be initially degraded into mono- and oligosaccharides through chemical, physical, and enzymatic methods [1, 4, 5]. Distinguished from other methods, enzymatic degradation is regarded as an environmental-friendly alternative. During the enzymatic degradation of mannans, β-mannanase (EC 3.2.1.78) is the most important enzyme among several mannan-degrading enzymes [6]. Thus far, the industrial applications of β-mannanases have developed rapidly in food, feed, and biorefinery industry [3, 7].

Based on the sequence similarities, β-mannanases belong to glycoside hydrolase (GH) families 5, 26, 113, and 134 in the CAZy database (carbohydrate-active enzymes database). So far, numerous β-mannanases from bacteria [8, 9], fungi [10, 11], and actinomyces [12, 13] have been discovered and characterized. Most of them belong to GH families 5 and 26 [14], while only a few β-mannanases are divided into GH families 113 and 134 [9, 11, 12]. Moreover, the β-mannanases from GH family 5 can be classified into subfamilies 7, 8, 10, and 17 [15]. In alkaline condition, β-mannanases from subfamily 8 display higher activity than subfamilies 7 and 10, which usually act at acidic and neutral pH [16]. Even though wild-type enzymes have various desirable properties, their biochemical properties also display many limitations, which make them not suitable for industrial applications in some cases [17]. After the pretreatments of biomass, such as acidic hydrolysis, hydrothermolysis, and steam explosion, the materials are usually tested to be acidic during biorefinery process [5, 18, 19]. Although many wild-type β-mannanases exhibit the maximal activity under acidic and/or thermophilic conditions, their specific activities maintain at low levels in their optimal pH and temperatures [20, 21], causing the high cost in production and then the limitation in biorefinery industry.

To satisfy the requirement of biorefinery application, β-mannanases can be engineered to optimize its biochemical properties. One of the most successful approaches is to engineer the enzyme via directed evolution [22]. Requiring little structure information of target protein, directed evolution has been increasingly exploited to optimize the traits of enzyme, such as optimal condition, stability, and selectivity [23–25]. However, only a few reports, so far, are available to improve β-mannanase properties via directed evolution. Wang et al. [26] have engineered a GH family 26 β-mannanase from *Pantoea agglomerans* A021, whose catalytic efficiency has increased at least 1.14-fold after directed evolution. In another study, the specific activities of two β-mannanases, belonging to GH families 5 and 26 from *Podospora anserina*, are significantly enhanced by directed evolution [27]. However, there is no information on change of the optimal conditions of β-mannanases via directed evolution yet.

*Rhizomucor miehei* CAU432 is a thermophilic filamentous fungus which can produce various glycoside hydrolase, such as endo-β-1,3-glucanase and lichenase [28, 29]. Its GH family 5 β-mannanase (*Rm*Man5A, GenBank Accession No. AGC24277.1) has been functionally and structurally characterized in previous reports [30, 31]. *Rm*Man5A exhibits a classical (β/α)_8_-TIM barrel fold and displays many outstanding properties, e.g., high specific activity and hydrolysis property, which makes it a suitable candidate for many industrial applications. However, *Rm*Man5A shows maximal activity at neutral pH and 50 °C, which largely limits its application in biorefinery. In this study, to enhance the catalytic activity of *Rm*Man5A in acidic and thermophilic conditions and gain more information on the structure–function relationship of β-mannanase, *Rm*Man5A was engineered using error-prone polymerase chain reaction (error-prone PCR), DNA shuffling, site-directed mutagenesis (SDM), and site-saturation mutagenesis (SSM). This strategy offers a potential to remodel the β-mannanase for increasing the catalytic efficiency under acidic and thermophilic conditions while retaining its specific activity and hydrolysis property. This is the first report on β-mannanase from a zygomycete engineered by directed evolution.

## Results

### Screening of the mutant library

**Fig. 1:**
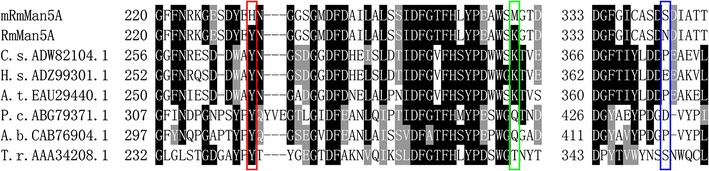
Amino acid sequence similarity of m*Rm*Man5A and *Rm*Man5A with other GH family 5 β-mannanases. *Numbers on the left* are the residue number of the first amino acid in each line. Sequences listed include the β-mannanases from *Chaetomium* sp. CQ31 (ADW82104.1), *Humicola* sp. Y1 (ADZ99301.1), *Aspergillus terreus* NIH2624 (EAU29440), *Phanerochaete chrysosporium* (ABG79371.1), *Agaricus bisporus* (CAB76904.1), and *Trichoderma reesei* (AAA34208.1). The single-letter amino acid code is used. Identical residues are shaded in *black*, and conserved residues are shaded in *gray*. The locations of three substitutions (Tyr233His, Lys264Met, and Asn343Ser) are *boxed* in *red*, *green*, and *blue*, respectively

A mutant library with a mutation frequency of 0.72% was generated by error-prone PCR and DNA shuffling using the primers in Additional file 1: Table S1. The summary of two rounds of screening and the number of mutants selected at each step are shown in Additional file 1: Figure S1. During the initial step of screening, the mutant library containing 12,000 clones were pre-screened by Congo red plate. Since the variant with improved activity at acidic pH was expected, the pH of screening plate was adjusted to 5.0 with McIlvaine buffer. More than 30% of the clones formed clear spots, while only 894 clones had clear halos whose diameter could be measured. The diameter of the control halo caused by *Rm*Man5A was about 4–5 mm. Compared to this size, only 198 clones showed similar or larger size of halos. The diameter of larger halos could reach 7–8 mm. Subsequently, the selected clones were cultured and inducted in 50-mL conical flasks and biochemical assays were conducted to analyze their optimal conditions. Finally, one mutant (m*Rm*Man5A) was obtained, displaying high catalytic activity under acidic and thermophilic conditions. According to the results of DNA sequencing, m*Rm*Man5A contained 5 base substitutions, two of which led to silent mutations (Ala261: gct-gcc, Leu318: tta-ttg), while the others led to three non-silent mutations, namely Tyr233His, Lys264Met, and Asn343Ser. Amino acid sequence alignment of *Rm*Man5A with the homologous β-mannanases was performed and revealed that residue Try233 was entirely conserved, Lys264 was partly conserved, and Asn343 was non-conserved in GH family 5 β-mannanases (Fig. 1).

### Expression and purification of protein

**Fig. 2:**
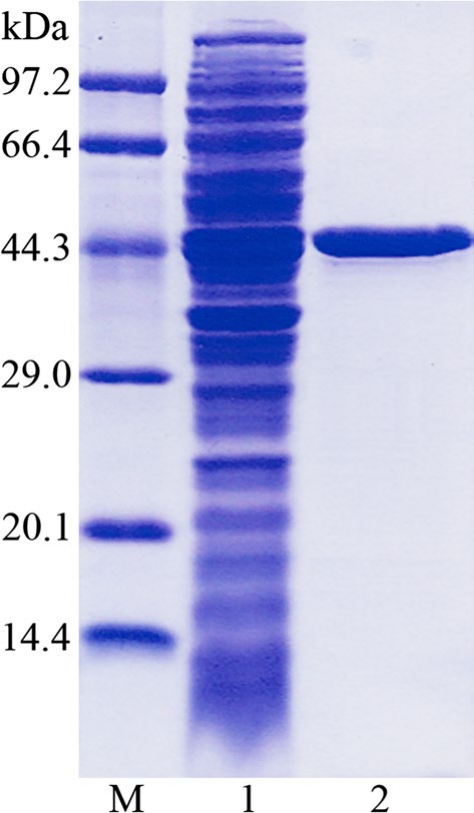
SDS-PAGE analysis of purified m*Rm*Man5. *Lane M* low molecular weight protein markers, *lane 1* crude lysate, *lane 2* purified m*Rm*Man5A

The mutant β-mannanase gene (*mRmMan5A*) was amplified by PCR and then inserted into pET28a (+) plasmid with *Bam*HI and *Xho*I restriction sites. Subsequently, the mutant β-mannanase without the signal peptide was successfully expressed in *Escherichia coli* BL21 and purified by Ni-IDA column chromatography with a purification fold of 8.2 and a recovery yield of 67.9%. Sodium dodecyl sulfate polyacrylamide gel electrophoresis (SDS-PAGE) analysis indicated that m*Rm*Man5A migrated as a single homogeneous band of 44 kDa (Fig. 2), which was same as *Rm*Man5A [30].

### Properties and kinetic parameters

**Fig. 3:**
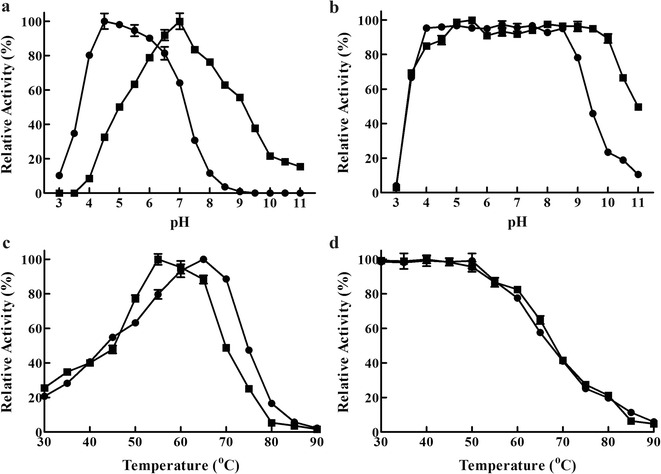
Biochemical properties of *Rm*Man5A (*filled square*) and m*Rm*Man5A (*filled circle*). **a** The effect of pH on β-mannanase activity in 50 mmol L^−1^ different buffers at 55 °C (for *Rm*Man5A) or 65 °C (for m*Rm*Man5A). **b** The pH stability of each enzyme in various pH ranges. **c** The influence of temperature on β-mannanase activity in 50 mmol L^−1^ McIlvaine buffer (pH 7.0 for *Rm*Man5A and pH 4.5 for m*Rm*Man5A). **d** The thermostability of each enzyme at different temperatures. For pH stability and thermostability, the residual activities of *Rm*Man5A were measured at pH 7.0 and 55 °C, and those of m*Rm*Man5A were measured at pH 4.5 and 65 °C. The specific activities of *Rm*Man5A (10,756.3 U mg^−1^) and m*Rm*Man5A (9671.4 U mg^−1^) were considered as 100% to determinate the optimal pH, pH stability, optimal temperature, and thermostability

Compared to *Rm*Man5A, optimal pH of m*Rm*Man5A was 4.5, which shifted 2.5 units towards acidic range (Fig. 3a). m*Rm*Man5A was found to exhibit more than 80% of the maximal activity after incubation at pH range of 4.0–8.5 for 30 min (Fig. 3b). Optimal temperature of m*Rm*Man5A was 65 °C, which was higher than that of *Rm*Man5A (55 °C) (Fig. 3c). m*Rm*Man5A showed excellent thermostability up to 55 °C (Fig. 3d), indicating that m*Rm*Man5A had inherited the thermostability of *Rm*Man5A.

Similar to *Rm*Man5A, m*Rm*Man5A showed the maximal specific activity towards locust bean gum (LBG, 9671.4 U mg^−1^), followed by konjac powder (5633.1 U mg^−1^) and guar gum (1472.1 U mg^−1^) (Table 1). The specific activities of m*Rm*Man5A were three times higher than those of *Rm*Man5A at pH 4.5 and 65 °C. Kinetic parameters of m*Rm*Man5A and *Rm*Man5A were measured with LBG and guar gum as substrates at pH 4.5 and 65 °C (Table 2). The maximal velocity (*V*_max_) of m*Rm*Man5A towards LBG was higher than that of *Rm*Man5A by 3.2-fold, while the *K*_m_ value of m*Rm*Man5A remained unchanged compared to *Rm*Man5A. Moreover, the catalytic efficiency (*k*_cat_/*K*_m_) of m*Rm*Man5A was enhanced about 3.3-fold at pH 4.5 and 65 °C. Similar results were obtained when guar gum was used as substrate.

**Table 1:** Substrate specificity of m*Rm*Man5A and *Rm*Man5A

Substrate	Specific activity (U mg^−1^; mean ± SD)		
	m*Rm*Man5A^a^	*Rm*Man5A^b^	
	pH 4.5, 65 °C	pH 4.5, 65 °C	pH 7.0, 55 °C
LBG	9671.4 ± 327	3011.8 ± 53	10,756.3 ± 424
Konjac powder	5633.1 ± 102	1382.6 ± 28	5317.6 ± 172
Guar gum	1472.1 ± 51	402.9 ± 5	1492.1 ± 27

^a^Enzymatic reactions were carried out at 65 °C for 10 min in 50 mmol L^−1^ McIlvaine buffer (pH 4.5)

^b^Enzymatic reactions were carried out at 65 °C for 10 min in 50 mmol L^−1^ McIlvaine buffer (pH 4.5) and at 55 °C for 10 min in 50 mmol L^−1^ McIlvaine buffer (pH 7.0), respectively

**Table 2:** Kinetic parameters of m*Rm*Man5A and *Rm*Man5A (mean ± SD)

Procedure	*V*_max_ (μmol min^‒1^ mg^−1^)	*K*_m_ (mg mL^‒1^)	*k*_cat_ (s^‒1^)	*k*_cat_/*K*_m_ (mg s^‒1^ mL^‒1^)
LBG				
m*Rm*Man5A	10,154.9 ± 293	3.27 ± 0.22	7446.9	2277.3
*Rm*Man5A	3192.5 ± 75	3.37 ± 0.18	2341.2	694.7
Guar gum				
m*Rm*Man5A	1516.3 ± 47	7.06 ± 0.42	1112.0	157.5
*Rm*Man5A	402.9 ± 11	7.12 ± 0.39	294.8	41.4

Enzymatic reactions were carried out in 50 mmol L^−1^ McIlvaine buffer (pH 4.5) at 65 °C for 5 min using LBG or guar gum as substrate

### Hydrolysis property

**Fig. 4:**
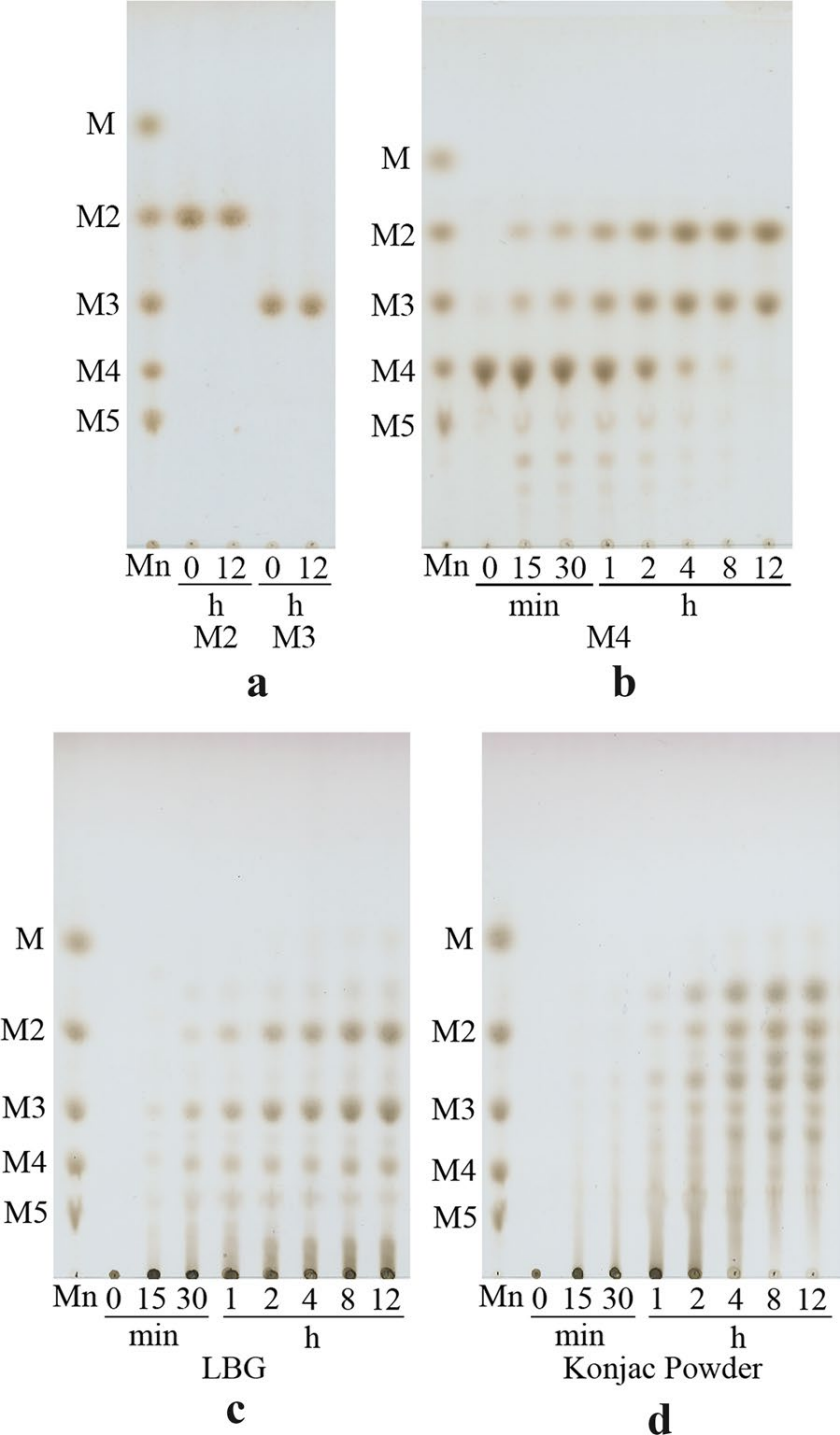
TLC analysis of mannans and manno-oligosaccharides hydrolysed by m*Rm*Man5A. m*Rm*Man5A (5 U mL^−1^) was incubated with 1% (w/v) mannobiose, mannotriose (**a**), mannotetrose (**b**), LBG (**c**), or konjac powder (**d**) in 50 mmol L^−1^ McIlvaine buffer (pH 4.5) at 50 °C for 12 h. Incubation times (hours or minutes) are indicated. *Lane Mn* standard of manno-oligosaccharides

The hydrolysis property of m*Rm*Man5A towards various manno-oligosaccharides and mannans was determined by TLC. m*Rm*Man5A could not degrade mannobiose (M2) and mannotriose (M3), even after prolonging the incubation period to 12 h (Fig. 4a). However, the enzyme could rapidly cleave mannotetrose (M4) to finally yield M2 and M3 (Fig. 4b). LBG was hydrolyzed by m*Rm*Man5A to yield predominantly M2, M3, and other manno-oligosaccharides with higher degree of polymerization (Fig. 4c). Due to the complexity of glucomannan in konjac powder, the hydrolysis product was a mixture containing M2, M3, and other unidentifiable oligosaccharides (Fig. 4d). Mannose (M) was not detected during the hydrolysis process of all substrates used.

### Hydrolysis of linear mannan

To compare the hydrolysis property of *Rm*Man5A and m*Rm*Man5A, the β-mannanases were employed to hydrolyze 5% (w/v) linear mannan at pH 4.5 and 50 °C. The concentration of manno-oligosaccharides was monitored during the hydrolysis (Additional file 1: Figure S2). After incubation with m*Rm*Man5A for 24 h, the concentration of M2 and M3 was markedly increased and reached 30.7 and 10.5 mg mL^−1^, respectively. In contrast, the hydrolysis by *Rm*Man5A was strongly inhibited, and the concentration of M2 and M3 only reached 11.2 and 3.1 mg mL^−1^, respectively.

### Site-directed mutagenesis

**Fig. 5:**
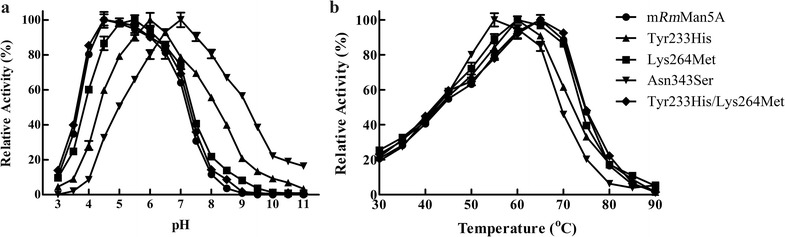
Determination of optimal pH (**a**) and optimal temperature (**b**) of m*Rm*Man5A and the variants derived using site-directed mutagenesis. The effect of pH on β-mannanase activity was determined in 50 mmol L^−1^ of different buffers at optimal temperature of each enzyme. For optimal temperature, activity was measured at different temperatures in 50 mmol L^−1^ McIlvaine buffer (optimal pH of each enzyme). The specific activities of m*Rm*Man5A (9671.4 U mg^−1^), variant Tyr233His (9834.6 U mg^−1^), variant Lys264Met (10,139.5 U mg^−1^), variant Asn343Ser (10,849.1 U mg^−1^), and variant Tyr233His/Lys264Met (9942.8 U mg^−1^) were considered as 100% in determinate the optimal pH and optimal temperature

To identify the effect of each substitution on biochemical property, three variants with single mutation (Tyr233His, Lys264Met, and Asn343Ser) were generated using SDM. Compared to *Rm*Man5A, optimal pH of variants Tyr233His and Lys264Met shifted 1 and 1.5 units towards the acidic range, respectively (Fig. 5a). Meanwhile, their optimal temperatures both increased by 5 °C (Fig. 5b). There was no change in optimal conditions between variant Asn343Ser and *Rm*Man5A (Fig. 5). Subsequently, the variant with double mutants (Tyr233His/Lys264Met) was generated and characterized. Its maximal activity was found in optimal condition of pH 4.5 and 65 °C (Fig. 5), which was same as m*Rm*Man5A. Moreover, the specific activities of four variants (9834.6, 10,139.5, 10,849.1, and 9942.8 U mg^−1^, respectively) showed no significant difference between *Rm*Man5A and m*Rm*Man5A at the optimal condition of each enzyme.

### Structure analysis of mutant enzyme

**Fig. 6:**
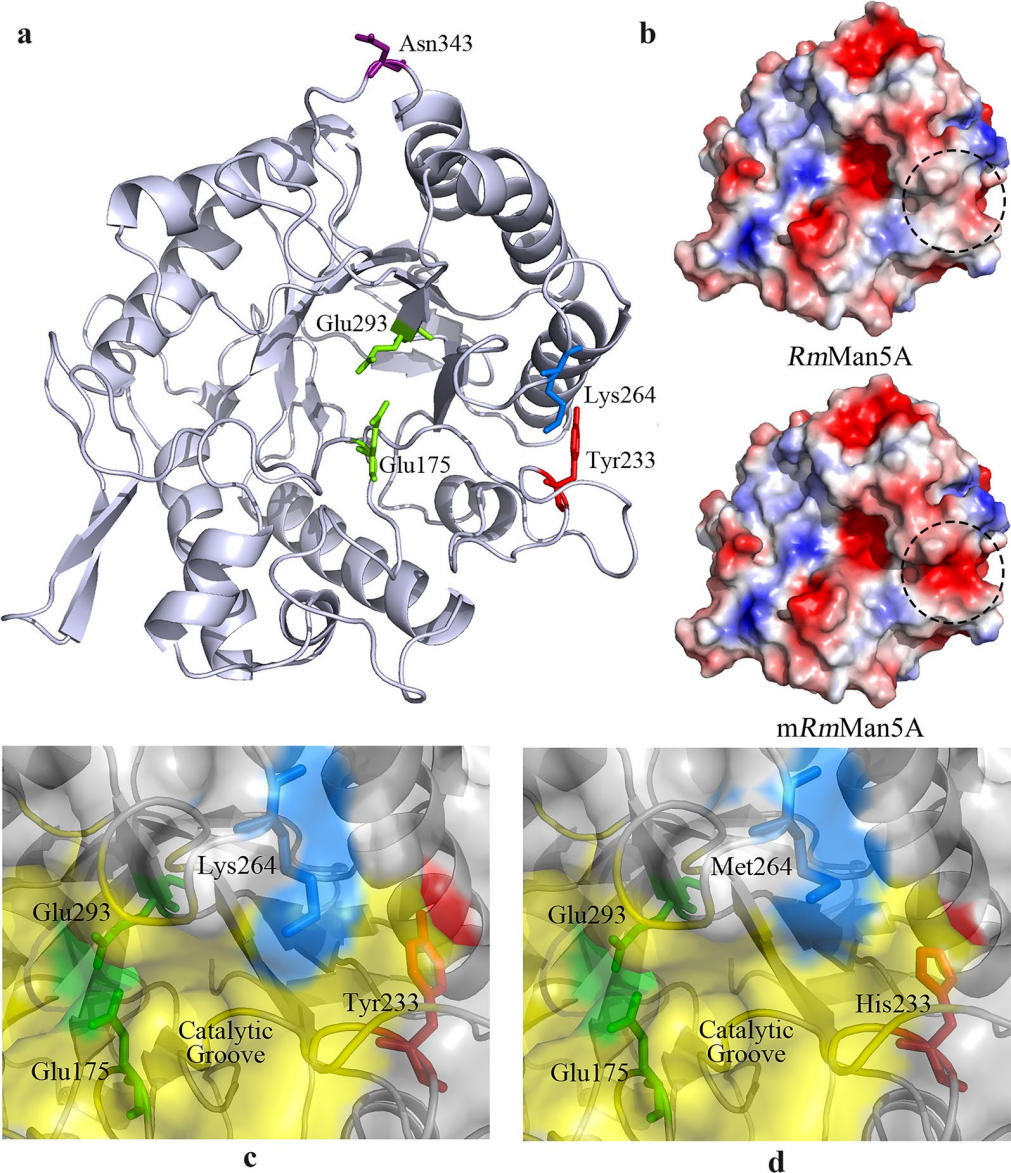
Localization of the three substitutions, surface view, and charge distribution in *Rm*Man5A and m*Rm*Man5A. **a** Localization of three residues (Tyr233, Lys264, and Asn343) and active site (Glu175 and Glu293) in *Rm*Man5A. **b** The protein surface charge of *Rm*Man5A and m*Rm*Man5A. The most negative and most positive electrostatic potentials are indicated by *red* and *blue*, respectively. **c** The location of Tyr233 and Lys264 in *Rm*Man5A before mutation. **d** The location of His233 and Met264 in m*Rm*Man5A after mutation. Tyr233, Lys264, and Asn343 were shown in *red*, *blue*, and *purple*, respectively. The active site and catalytic groove were colored in *green* and *yellow*, respectively

As a GH family 5 β-mannanase, *Rm*Man5A shares less than 40% of amino acid identities with other structurally determined β-mannanases and its overall structure exhibits a classical (β/α)_8_-TIM barrel fold [31]. According to the structure of *Rm*Man5A, all three substitutions (Tyr233His, Lys264Met, and Asn343Ser) were located on the surface of enzyme (Fig. 6a). In *Rm*Man5A, the distances between Tyr233, Lys264, and the catalytic amino acids were 16 and 14 Å, respectively. In contrast, the residue Asn343 was much far away from the catalytic site (27‒30 Å). Moreover, charge distribution of the right area of catalytic groove was transferred after directed evolution (Fig. 6b).

### Site-saturation mutagenesis

To further investigate the effect of residues Tyr233 and Lys264 on the enzymatic properties of *Rm*Man5A, two SSM libraries at residues Tyr233 and Lys264 were generated. All twenty variants of each position were collected after sequencing about 100 clones of each library. Optimal pH and temperatures of all variants were measured (Additional file 1: Table S2). Among the twenty variants of Tyr233, none of the variants showed changes in optimal conditions except variant Tyr233His (pH 6.0 and 60 °C), Tyr233Arg (pH 6.5 and 55 °C), and Tyr233Ile (pH 6.5 and 55 °C). With respect to Lys264, many substitutions could decrease the pH optimum of *Rm*Man5A, e.g., Lys264Gly, Lys264Val, Lys264Glu, and Lys264Asp, but none of them changed the optimal temperature of *Rm*Man5A. In contrast, variant Lys264Cys displayed the lowest optimal pH (pH 5.0 instead of 7.0 for *Rm*Man5A and pH 5.5 for variant Lys264Met) and the highest optimal temperature (60 °C, same with Lys264Met). To reveal the interplay of these two substitutions, the combinatorial mutagenesis was also generated. Variant Tyr233His/Lys264Cys showed the maximal activity at pH 4.5 and 65 °C. Moreover, the specific activity of variant Tyr233His/Lys264Cys was 9878.8 U mg^−1^, which was not influenced after mutagenesis compared to *Rm*Man5A and m*Rm*Man5A.

## Discussion

Owing to the various biochemical properties, β-mannanases are widely used in many industrial applications, including biorefinery [7]. However, wild-type enzymes often show defects in biochemical properties, which limit their application in some industries [17]. Directed evolution is usually regarded as an effective means to improve the biochemical properties of enzyme, even when lack of structure information impedes the use of rational design [17]. The GH family 5 β-mannanase (*Rm*Man5A) from *R. meihei* CAU432 shows high specific activity and can hydrolyze many mannan substrates into manno-oligosaccharides, which makes this β-mannanase a suitable candidate for many industry applications [30]. However, its catalytic activity is significantly low in acidic and thermophilic conditions, which are common in biorefinery industry. In this study, to enhance its catalytic efficiency under acidic and thermophilic conditions and to improve its feasibility in biorefinery application, *Rm*Man5A was engineered by directed evolution.

After error-prone PCR and DNA shuffling, a mutant library was generated with a gene mutation frequency of 0.72%, which is higher than the recommended mutation ratio (2–5 bp/kp) [27]. Therefore, about 70% clones of the mutant library were inactive clones. Eventually, one mutant β-mannanase (m*Rm*Man5A) with three substitutions (Tyr233His, Lys264Met, and Asn343Ser) was discovered through two rounds of screening. Optimal pH of m*Rm*Man5A was shifted from pH 7.0 to 4.5 (Fig. 3a), and optimal temperature of m*Rm*Man5A was improved from 55 to 65 °C (Fig. 3c). After directed evolution, the optimal pH of m*Rm*Man5A is much lower than those of many wild-type β-mannanases from *Bacillus subtilis* (pH 6.5) [32], *Myceliophthora thermophila* (pH 6.0) [33], and *Thermobifida fusca* (pH 8.0) [34]. Although the optimal condition of m*Rm*Man5A still can not reach the level of several wild-type β-mannanases, such as rMan5P1 (pH 4.0, 80 °C) [20], Man5A2 (pH 4.0, 90 °C) [21], and MAN-P (pH 4.5, 80 °C) [35], the specific activities of these β-mannanases are significantly lower than that of m*Rm*Man5A at their optimal conditions (Table 1). After directed evolution, the mutant inherited the specific activity of *Rm*Man5A, which is even higher than some wild-type β-mannanases with high specific activity, including the enzymes from *B. subtilis* (7302 U mg^−1^ towards LBG) [32] and *Enterobacter* sp. (8132 U mg^−1^ towards LBG) [36]. In the optimal condition, the specific activity of m*Rm*Man5A was significantly enhanced more than three times compared to *Rm*Man5A (Table 1), and the catalytic efficiency was remarkably improved with *k*_cat_/*K*_m_ value being at least 328% higher than that of the wild-type (Table 2). These changes indicate that the catalytic activity of *Rm*Man5A has been enhanced in acidic and thermophilic conditions. Moreover, there was no change between the *K*_m_ values of m*Rm*Man5A and *Rm*Man5A, indicating that the affinity for substrates is not influenced by directed evolution. In this study, main biochemical properties of m*Rm*Man5A, namely the optimal pH and temperature, were enhanced in one mutant at the same time. Similar phenomenon is only observed in few enzymes, such as feruloyl esterase and xylanase [37–39]. Furthermore, m*Rm*Man5A also inherited the excellent hydrolysis property of *Rm*Man5A. When LBG and konjac powders were hydrolyzed by m*Rm*Man5A, the polysaccharides were rapidly degraded into M2, M3, and other oligosaccharides (Fig. 4). When linear mannan was hydrolyzed by m*Rm*Man5A at pH 4.5 and 50 °C for 24 h, the concentration of M2 and M3 yielded was much higher than the values obtained by *Rm*Man5A (Additional file 1: Figure S2). This indicates that the improved biochemical property is valuable to enhance the application of m*Rm*Man5A in mannan degradation in acidic and thermophilic conditions. The comprehensive properties obtained by directed evolution make m*Rm*Man5A a potential candidate for biorefinery of mannan biomasses, which is conducted in acidic and thermal environments.

In general, most key mutations which lead to the evolution of properties, such as catalytic efficiency, specific activity, and optimal condition, are located on the surface of enzyme [24, 26, 40]. Conforming to this, all three substitutions (Tyr233His, Lys264Met, and Asn343Ser) were situated on the surface of *Rm*Man5A (Fig. 6a). To determine the influence of each amino acid substitution, three variants (Tyr233His, Lys264Met, and Asn343Ser) were generated by SDM. Among these variants, variant Tyr233His displayed noticeable changes with the optimal condition of pH 6.0 and 60 °C (Fig. 5). In *Rm*Man5A, Tyr233 with high hydrophobic feature was located near the catalytic groove, and its hydrophobic side chain pointed to the α-helix six (Fig. 6c). It is generally assumed that hydrophobic residues are not favorable at similar sites for protein stability [41, 42]. In variant Tyr233His, His with a hydrophilic side chain was inserted by substitution Tyr233His (Fig. 6d). This replacement increases the hydrophilic nature of the surface, and was found to stabilize the protein when compared with *Rm*Man5A. Moreover, the number of polar residues (Asn, His, and Gln) in acidic β-mannanases is normally more than that in alkaline β-mannanases [16]. This might be another reason for the change of variant Tyr233His. Variant Lys264Met was another significant variant, which showed acidic shift by 1.5 units of optimal pH and increase by 5 °C of optimal temperature (Fig. 5). In *Rm*Man5A, the distance between Lys264 and the catalytic site was 14 Å. Normally, the ionizable residues in the vicinity of catalytic residues have the ability to affect the pH-dependent activity of enzyme or even the catalysis process to some extent [26, 43]. In variant Lys264Met, the positively charged residue Lys264 on the edge of catalytic groove was substituted by a non-polar residue Met (Fig. 6d). This substitution affects the local charge balance near the catalysis groove and improves the catalytic efficiency of mutant in acidic condition. In contrast, Asn343 was much further (27–30 Å) from the catalytic amino acids. Thus, variant Asn343Ser showed imperceptible changes compared to *Rm*Man5A (Fig. 5). To determine the interactive effect of Tyr233His and Lys264Met, the variant with double mutations was generated. Optimal conditions of variant Tyr233His/Lys264Met were same as m*Rm*Man5A (Fig. 5), which means that the combination of Tyr233His and Lys264Met is the major reason for property evolution of m*Rm*Man5A. According to the analysis of protein surface charge (Fig. 6b), weak negative charge in the right area of catalytic groove of *Rm*Man5A was transformed into strong negative charge after directed evolution. This change mainly caused by Tyr233His and Lys264Met. In general, the charge change of catalytic groove can impact on the substrate acceptance in the catalytic groove [40, 42]. However, the *K*_m_ values of m*Rm*Man5A did not show significant change comparing to that of *Rm*Man5A after directed evolution. This means that the substrate acceptance of m*Rm*Man5A is not influenced by the charge change. The improvement of catalytic efficiencies (*k*_cat_/*K*_m_) in acidic and thermophilic condition is originated from the increase of *V*_max_ and *k*_cat_ of m*Rm*Man5A. This may be similar reason for the catalytic efficiency increase of many other enzymes such as phytase and protease [44, 45]. Moreover, SSM on residues Tyr233 and Lys264 of *Rm*Man5A were performed. Similar to the result of SDM, residue Tyr233 showed less influence on the enzymatic properties. Only variant Tyr233His showed noticeable changes in optimal conditions. The substitution of Lys264 is easier to affect the enzymatic properties than that of Tyr233. Among the twenty variants, four variants (Lys264Gly, Lys264Val, Lys264Glu, and Lys264Asp) have acidic shift in pH optima, and the variant Lys264Cys displayed significant change in both of optimal pH and temperature. The pH optimum of variant Lys264Cys is even lower than variant Lys264Met. However, the variant with double mutant (Tyr233His/Lys264Cys) did not exhibit any improvement in optimal conditions compared to m*Rm*Man5A. According to the result of sequence alignment (Fig. 1), the residues Try233 and Lys264 are entirely or partly conserved in GH family 5 β-mannanases. Therefore, these results can provide some basis for the engineering and modification of GH family 5 β-mannanase in future.

## Conclusions

A β-mannanase (*Rm*Man5A) from *R. meihei* CAU432 was engineered by directed evolution. The mutant β-mannanase (m*Rm*Man5A) showed an improved catalytic activity under acidic and thermophilic conditions without any significant change in other properties of *Rm*Man5A. m*Rm*Man5A can be a suitable candidate for biorefinery of mannan biomasses, which commonly needs acidic and thermophilic environment. Two substitutions (Tyr233His and Lys264Met) were the main reason for the property evolution of m*Rm*Man5A. Some new information on the structure–function relationship of β-mannanase was provided for the engineering and modification of GH family 5 β-mannanase.

## Methods

### Vectors and chemicals

The pET28a-*RmMan5A* was constructed as described previously [30]. *E. coli* DH5α and BL21 (DE3) were used for plasmids propagating and gene expression, respectively. Vector pET28a (+) was obtained from Novagen (Madison, WI, USA). Chelating Sepharose (Ni-IDA) resin matrix was purchased from GE Life Sciences (Pittsburgh, PA, USA). LBG and guar gum were obtained from Sigma (St. Louis, MO, USA). Unless otherwise stated, all other chemicals were of analytical grade.

### Generation of the mutant library

DNA shuffling mutant library was generated according to previous description with slight modifications [40]. Random libraries were constructed by error-prone PCR using pET28a-*RmMan5A* as template with primers *Rm*Man5AF and *Rm*Man5AR (Additional file 1: Table S1). The amplification products were digested by DNase I according to Lorimer and Pastan [46]. The full-length gene was reassembled by self-reassembling PCR using the purified DNA fragments. After digestion with *Bam*HI and *Xho*I, PCR products were ligated with plasmid pET-28a (+). The resulting recombinant plasmids were transformed into *E. coli* BL21 (DE3) to build the mutant library.

### Screening of the mutant library

In first round of selection, Congo red plate assay was used to screen the forward mutants. All mutants were spread on a Luria–Bertani (LB) plate, containing 50 μg mL^−1^ kanamycin, and incubated at 37 °C for 1 day. Every single colony was then transferred into the screening LB plates using sterile toothpick, which contained 50 mmol L^−1^ McIlvaine buffer (pH 5.0), 1 mmol L^−1^ IPTG, 0.5% (w/v) LBG, and 50 μg mL^−1^ kanamycin. As a control, the wild-type *Rm*Man5A was inoculated on each plate. After incubation at 37 °C for 24 h, the cells were lysed at 60 °C for 1 h. Subsequently, all plates were stained by 0.1% (w/v) Congo red and washed by 1 mol L^−1^ NaCl until the halos appeared. The size of halos was measured and compared to the control. Transformants with similar or larger halo were selected for next selection. In second round of selection, the optimal conditions of selected transformant were measured, and the mutants with change on optimal conditions were then sequenced.

### Expression and purification of the mutants

The enzyme was expressed and purified as previously described [30]. Briefly, after induction with 1% (w/v) lactose for 20 h, the cells were harvested and then disrupted by sonication. The crude enzyme obtained through centrifugation was applied to a Ni-IDA agarose resin column and then purified by fast protein liquid chromatography (FPLC, ÄKTA purifier, GE healthcare). Impurities were washed off with five column volumes of buffer A (20 mmol L^−1^ phosphate, 300 mmol L^−1^ NaCl, and 20 mmol L^−1^ imidazole at pH 8.0) and buffer B (20 mmol L^−1^ phosphate buffer, 300 mmol L^−1^ NaCl, and 50 mmol L^−1^ imidazole at pH 8.0). The bound protein was then eluted with buffer C (20 mmol L^‒1^ phosphate buffer, 300 mmol L^−1^ NaCl, and 200 mmol L^−1^ imidazole at pH 8.0). Fractions showing β-mannanase activity were pooled and concentrated by ultrafiltration. SDS-PAGE was executed to analyze the purity and molecular mass on a 12.5% gel [47].

### Enzyme assay and protein concentration

To determine the β-mannanase activity, the amount of reducing sugar was determined using 3,5-dinitrosalicylic acid (DNS) method [48]. A total of 0.1 mL of diluted enzyme was mixed with 0.9 mL of LBG solution (0.5%, w/v, 50 mmol L^−1^ McIlvaine buffer, pH 4.5) and incubated at 65 °C for 10 min. After addition with 1 mL of DNS, the mixture was boiled for 15 min, and immediately added with 1 mL of 40% (w/v) sodium potassium tartarate. The absorbance was measured at 540 nm and mannose was used as standard for reducing sugar content calculation. One unit of β-mannanase activity was defined as the amount of enzyme that liberated 1 μmol of reducing sugars per minute under the conditions described. The concentration of protein was measured by Lowry’s method using bovine serum albumin as standard [49].

### Biochemical properties and kinetic parameters

Optimal pH of enzyme was determined in 50 mmol L^−1^ different buffers with pH ranging from 3.0 to 11.0. The buffers included McIlvaine (pH 3.0‒7.0), acetate (pH 4.0–6.0), phosphate (pH 6.0–8.0), tris (hydroxymethyl) aminomethane-HCl (Tris–HCl) (pH 7.0–9.0), 2-(cyclohexylamino) ethanesulfonic acid (CHES) (pH 8.0–10.0), and glycine-NaOH (pH 9.5–11.0). To measure pH stability, enzyme samples were incubated in above buffers at 50 °C for 30 min, and the residual activities were determined by standard assay. Optimal temperature was assessed in 50 mmol L^−1^ McIlvaine buffer (pH 4.5) at a temperature range of 30–90 °C. Effect of temperature on enzyme stability was examined by determining the residual activity after incubating at different temperatures for 30 min.

Substrate specifities of β-mannanase towards 0.5% (w/v) of LBG, konjac powder, and guar gum were measured at 65 °C and pH 4.5. Kinetic parameters toward LBG and guar gum were determined at the substrates concentration ranges of 1–7 and 3–14 mg mL^−1^, respectively, at 65 °C in 50 mmol L^−1^ McIlvaine buffer (pH 4.5) for 5 min using DNS method. The value of *K*_m_ and *V*_max_ was calculated using the software GraFit. All activity assays were performed in triplicate.

### Hydrolysis property

Hydrolysis properties of m*Rm*Man5A towards various manno-oligosaccharides and mannans were analyzed as given below. A total of 1 mL of reaction mixture containing 1% (w/v) of LBG, konjac power, M2, M3, or M4 and 5 U of m*Rm*Man5A was incubated in 50 mmol L^−1^ McIlvaine buffer (pH 4.5) at 50 °C for 12 h. Samples of different time points were withdrawn and boiled for 5 min. After centrifugation at 10,000×*g* for 3 min, all samples were analyzed by thin-layer chromatography (TLC). The silica gel plate (Merck Silica Gel 60 F254, Darmstadt, Germany) loaded with samples was developed twice in a solvent system, containing n-butylalcohol:acetic acid:water (2:1:1, v/v). After immersing in the solution (methanol:sulfuric acid 95:5, v/v), the plate was heated in an oven to visualize the saccharides. Mixtures of M-M4, and mannopentaose (M5) were used as standards.

### Hydrolysis of linear mannan

*Rm*Man5A and m*Rm*Man5A were subjected to hydrolyze linear mannan. Briefly, 1.0 g of linear mannan was mixed with 20 mL 50 mmol L^−1^ McIlvaine buffer (pH 4.5). Then the β-mannanase was added in a proportion of 50 U mL^−1^ and enzymatic reaction was performed at 50 °C and 200 rpm for 24 h. Samples of different time points were withdrawn and boiled for 5 min. All samples were analyzed by high performance liquid chromatography (HPLC, Waters Alliance 2695 system) equipped with evaporative light-scattering detector (ELSD, Waters 2424 HPLC ELS). The separation of manno-oligosaccharides was completed on a Sugar KS-802 column (7.8 × 300 mm, Shodex) with ultrapure water as mobile phase at a flow rate of 0.8 mL min^−1^. Column temperature was maintained at 80 °C. Nitrogen was used as nebulising gas for ELSD at a flow rate 2.0 L min^−1^ and the temperature of drift tube was fixed at 50 °C. M-M5 were used as standards.

### Site-directed mutagenesis

The overlap extension PCR method was used to perform SDM as previously described [24]. The plasmid pET28a-*RmMan5A* was used as template and the primers were designed according to three substitutions (Additional file 1: Table S1). The products were ligated to plasmid pET-28a (+) after digestion with *Bam*HI and *Xho*I. Then the plasmids harboring the desired mutations were transformed into *E. coli* BL21 (DE3) for expression.

### Site-saturation mutagenesis

SSM on residues Try233 and Lys264 of *Rm*Man5A was carried out as described in previous literature [50]. The plasmid pET28a-*RmMan5A* was used as template and the primers were designed using NNK degeneracy (Additional file 1: Table S1). The PCR products were digested by *Dpn*I overnight at 37 °C, and the mixture was then incubated at 80 °C for 20 min to inactivate *Dpn*I. After purification, the products were transformed into *E. coli* BL21 (DE3) competent cells by electroporation transformation. The transformants were subsequently cultured on LB plate at 37 °C overnight. The DNA sequences of single colonies were sequenced to collect all 20 variants of each position.

### Structure analysis of mutant enzyme

The 3D structure of *Rm*Man5A had been reported in our previous work (PBD ID: 4qp0) [31]. Visualization and analysis of structural model were performed using PyMOL software.

## Additional file

**Additional file 1: Table S1.** Oligonucleotide primers used for plasmid construction, site-directed mutagenesis, and site-saturation mutagenesis. **Table S2**. Optimal pH and temperature of twenty variants of Tyr233 and Lys264. **Figure S1**. The summary of screening strategy and mutant selection. **Figure S2**. HPLC analysis of linear mannan hydrolyzed by m*Rm*Man5A and *Rm*Man5A.

## Abbreviations

GH: glycoside hydrolase
error-prone PCR: error-prone polymerase chain reaction
SDM: site-directed mutagenesis
SSM: site-saturation mutagenesis
Ni-IDA: Ni-iminodiacetic acid
SDS-PAGE: sodium dodecyl sulfate polyacrylamide gel electrophoresis
LBG: locust bean gum
M: mannose
M2: mannobiose
M3: mannotriose
M4: mannotetrose
M5: mannopentaose
DNS: 3,5-dinitrosalicylic acid
Tris: tris (hydroxymethyl) aminomethane
CHES: 2-(cyclohexylamino) ethanesulfonic acid
TLC: thin-layer chromatography
