# Supplementary material for: Directed evolution of a β-mannanase from Rhizomucor miehei to improve catalytic activity in acidic and thermophilic conditions
Source: Biotechnol Biofuels. 2017 Jun 2;10:143. doi: 10.1186/s13068-017-0833-x (PMC5457547; doi:10.1186/s13068-017-0833-x)
Supplement: Supplementary file 1 — Additional file 1: Table S1. Oligonucleotide primers used for plasmid construction, site-directed mutagenesis, and site-saturation mutagenesis. Table S2. Optimal pH and temperature of twenty variants of Tyr233 and Lys264. Figure S1. The summary of screening strategy and mutant selection. Figure S2. HPLC analysis of linear mannan hydrolyzed by mRmMan5A and RmMan5A. [file 13068_2017_833_MOESM1_ESM.doc]

**Supplementary Information**

**Directed evolution of a β-mannanase from *Rhizomucor miehei* to improve catalytic activity in acidic and thermophilic conditions**

**Yanxiao Li^1^, Ping Yi****^1^, Qiaojuan Yan^1*^****, Zhen Qin^2^, Xueqiang Liu^1^, Zheng-qiang Jiang^1*^**

^1^ Beijing Advanced Innovation Center for Food Nutrition and Human Health, Bioresource Utilization Laboratory, College of Engineering, China Agricultural University, Beijing, China

^2^ Beijing Advanced Innovation Center for Food Nutrition and Human Health, College of Food Science and Nutritional Engineering, China Agricultural University, Beijing, China

Yanxiao Li, e-mail: liyanxiao2012@cau.edu.cn

Ping Yi, e-mail: yiping5129@126.com

Zhen Qin, e-mail: qzh1026@foxmail.com

Xueqiang Liu, e-mail: caulxq@163.com

***** Correspondence. Tel.: +86 10 62737689; fax: +86 10 82388508. e-mail: zhqjiang@cau.edu.cn (Z.Q. Jiang); yanqj@cau.edu.cn (Q.J. Yan)

Address for correspondence: Professor Drs. Zhengqiang Jiang & Qiaojuan Yan

Post Box 294, China Agricultural University, No.17 Qinghua Donglu, Haidian District, Beijing 100083, China

**Table S1** Oligonucleotide primers used for plasmid construction, site-directed mutagenesis, and site-saturation mutagenesis.

| Primers | Sequence (5′→3′) |
| --- | --- |
| *Rm*Man5AF *^a^* | CGCGGATCCGCTTCTTCGTTTGTCCAGACAAG |
| *Rm*Man5AR *^a^* | CCGCTCGAGCTACTTCTTGGCCATGGCATCAGC |
| Tyr233HisF | GGAGAATCCGACTACGAACACAACGGTGG |
| Tyr233HisR | GTTCGTAGTCGGATTCTCCTTTGCGGTTAAAG |
| Lys264MetF | ACCCTGAAGCTTGGAGCATGGGTACCGATTC |
| Lys264MetR | ATGCTCCAAGCTTCAGGGTAAAGATGGAAAGTG |
| Asn343SerF | GAATTTGCGCCTCAGATAGCGATATTGCTAC |
| Asn343SerR | CTATCTGAGGCGCAAATTCCAAAGCCATCC |
| SSM233F ^b^ | GGAGAATCCGACTACGAANNKAACGGTGGATCGG |
| SSM233R ^b^ | CCGATCCACCGTTMNNTTCGTAGTCGGATTCTCC |
| SSM264F ^b^ | CCCTGAAGCTTGGAGCNNKGGTACCGATTCG |
| SSM264R ^b^ | CGAATCGGTACCMNNGCTCCAAGCTTCAGGG |

*^a^* Incorporated restriction enzyme sites are underlined.

^b^ N is A, G, C, or T; K is G or T; M is A or C.

**Table S2** Optimal pH and temperature of twenty variants of Tyr233 and Lys264.

| Residue | Optimal pH and temperature ^a^ | |
| --- | --- | --- |
|  | Tyr233 | Lys264 |
| Tyr ^b^ | 7.0, 55 °C | 7.0, 55 °C |
| Lys ^b^ | 7.0, 55 °C | 7.0, 55 °C |
| His ^c^ | 6.0, 60 °C | 7.0, 55 °C |
| Met ^c^ | 7.0, 55 °C | 5.5, 60 °C |
| Ala | 7.0, 55 °C | 7.0, 55 °C |
| Thr | 7.0, 55 °C | 7.0, 55 °C |
| Gly | 7.0, 55 °C | 6.5, 55 °C |
| Leu | 7.0, 55 °C | 7.0, 55 °C |
| Pro | 7.0, 55 °C | 7.0, 55 °C |
| Val | 7.0, 55 °C | 6.5, 55 °C |
| Ser | 7.0, 55 °C | 7.0, 55 °C |
| Cys | 7.0, 55 °C | 5.0, 60 °C |
| Arg | 6.5, 55 °C | 7.0, 55 °C |
| Glu | 7.0, 55 °C | 6.5, 55 °C |
| Asp | 7.0, 55 °C | 6.5, 55 °C |
| Phe | 7.0, 55 °C | 7.0, 55 °C |
| Ile | 6.5, 55 °C | 7.0, 55 °C |
| Asn | 7.0, 55 °C | 7.0, 55 °C |
| Gln | 7.0, 55 °C | 7.0, 55 °C |
| Trp | 7.0, 55 °C | 7.0, 55 °C |

^a^ The optimal pH of each variant was measured in 50 mmol L^‒1^ of different buffers at its optimal temperature, and the optimal temperature was determined at different temperature in 50 mmol L^‒1^ McIlvaine buffer (optimal pH of each variant).

^b^ The origin residue of the wild type β-mannanase (*Rm*Man5A).

^c^ The mutant residue of the mutant β-mannanase (m*Rm*Man5A).





**Fig. S1** The summary of screening strategy and mutant selection.

**
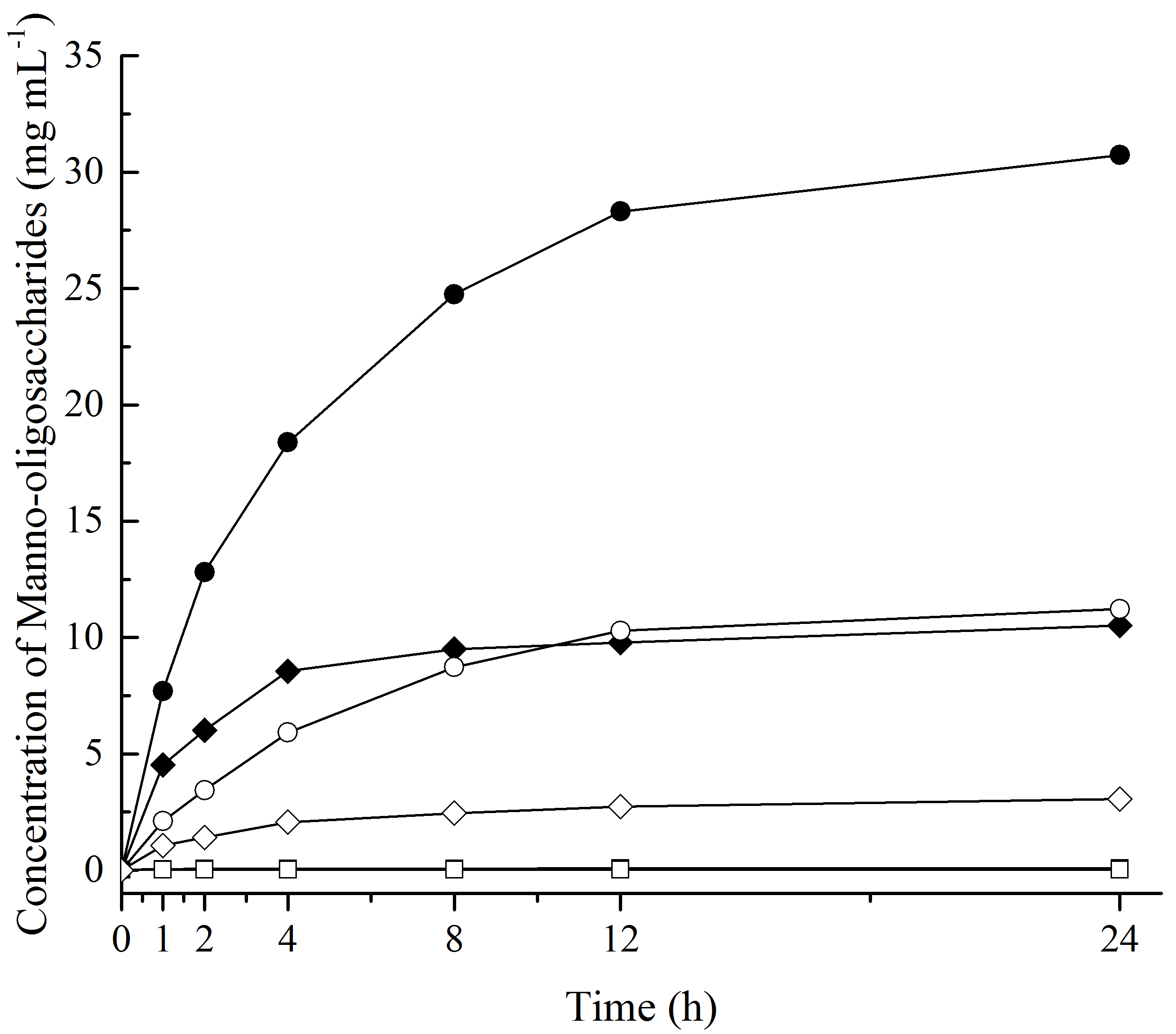
**

**Fig. S2** HPLC analysis of linear mannan hydrolyzed by m*Rm*Man5A (A) and *Rm*Man5A (B). Linear mannan (5%, w/v) was hydrolyzed by m*Rm*Man5A or *Rm*Man5A (50 U mL^‒1^), respectively, in 50 mmol L^‒1^ McIlvaine buffer (pH 4.5) at 50 °C for 0, 1, 2, 4, 8, 12, and 24 h. M (■), M2 (●), and M3 (◆) produced by m*Rm*Man5A were quantified using HPLC-ELSD. M (□), M2 (○), and M3 (◇) were produced by *Rm*Man5A.
